# Liposomes for effective drug delivery to the ocular posterior chamber

**DOI:** 10.1186/s12951-019-0498-7

**Published:** 2019-05-13

**Authors:** Sisi Lai, Yanyan Wei, Quanwu Wu, Kang Zhou, Tuo Liu, Yingfeng Zhang, Ning Jiang, Wen Xiao, Junjie Chen, Qiuhong Liu, Yang Yu

**Affiliations:** 1 School of Pharmaceutical Sciences, Guangzhou University of Chinese Medicine, Guangzhou, 510006 Guangdong China; 2 The First Affiliated Hospital, Guangzhou University of Chinese Medicine, Guangzhou, 510006 Guangdong China

**Keywords:** PAMAM G3.0-coated compound liposomes, Ocular drug delivery, Berberine hydrochloride, Chrysophanol, Transcorneal permeability, Antioxidative retinal damage

## Abstract

**Background:**

Age-related macular degeneration (AMD) is a leading cause of severe visual deficits and blindness. Meanwhile, there is convincing evidence implicating oxidative stress, inflammation, and neovascularization in the onset and progression of AMD. Several studies have identified berberine hydrochloride and chrysophanol as potential treatments for ocular diseases based on their antioxidative, antiangiogenic, and anti-inflammatory effects. Unfortunately, their poor stability and bioavailability have limited their application. In order to overcome these disadvantages, we prepared a compound liposome system that can entrap these drugs simultaneously using the third polyamidoamine dendrimer (PAMAM G3.0) as a carrier.

**Results:**

PAMAM G3.0-coated compound liposomes exhibited appreciable cellular permeability in human corneal epithelial cells and enhanced bio-adhesion on rabbit corneal epithelium. Moreover, coated liposomes greatly improved BBH bioavailability. Further, coated liposomes exhibited obviously protective effects in human retinal pigment epithelial cells and rat retinas after photooxidative retinal injury. Finally, administration of P-CBLs showed no sign of side effects on ocular surface structure in rabbits model.

**Conclusions:**

The PAMAM G3.0-liposome system thus displayed a potential use for treating various ocular diseases.

**Electronic supplementary material:**

The online version of this article (10.1186/s12951-019-0498-7) contains supplementary material, which is available to authorized users.

## Background

More than 5% people worldwide suffer visual impairment caused by AMD, which induced heavy burden with huge economic cost more than 300 billion dollar [1] and the global prevalence of AMD is likely to rise as a consequence of the aging population [2]. However, the physiological and anatomical structure of the eye represents great challenges for developing effective therapies for AMD.

In order to overcome these barriers, different therapeutic strategies have been developed for the treatment of AMD. As therapeutic drugs delivered via intravitreal injection can reach the target tissue directly at a therapeutic concentration, this is regarded as the most essential strategy for treating AMD, particularly wet AMD [3]. However, this approach has some limitations, including repeated-injection-induced complications such as inflammation, vitreous hemorrhage, retinal detachment, and endophthalmitis [4–6], also resulting in significant side effect on serum or plasma level after intravitreal injection of anti-VEGF [7, 8]. Meanwhile, advancements in the field of nanotechnology such as nanoparticles [9], micellar carrier [10] and dendrimer [11] have emerged, displaying effective accumulation in the cornea via noninvasive routes and providing controlled release with minimal side effects.

Liposomes, which are nanocarriers consisting of a cellular-membrane-like lipid bilayer surrounding an aqueous phase, represent a promising avenue for ocular drug delivery based on their various advantages, such as an increased residence time for drug absorption [12]. In addition, their half-lives in vitreous bodies can be prolonged with low toxicity [13]. However, their entry into deeper tissues is limited because of their low bioadhesion. Currently, a variety of biomacromolecules, such as polymers [14] and specific ligand [15] have been used along with liposomes for enhancing transshipment efficiency. PAMAM G3.0, the third-generation formulation of polyamidoamine dendrimer, characterized by a high degree of dendritic branches and high-density functional amino groups, provides an excellent platform for drug-delivery systems because of its advantages, including high drug loading, high water-bonding capacity, and low toxicity [16–18]. In addition, its spherical shape and enormous internal hydrophobic cavities allow it to encapsulate drug molecules similarly as micelles, which could also modify the liposomal structure to facilitate targeting [19–22].

Berberine hydrochloride (BBH), an active ingredient of Rhizoma Coptidis, has been widely employed in the treatment of gastrointestinal disorders in China owing to its diverse pharmacologic activities. Recently, it has gained increasing attention because of its potential biological activities, including antioxidant [23, 24], anti-inflammatory [25, 26], and antidiabetic [26–28] activities, as well as its significant inhibition of vascular smooth muscle cell proliferation [25, 29]. Chrysophanol (CHR), an anthraquinone extracted from the Chinese herbs *Rheum palmatum* L. and *Rheum officinale* Baill., is used to treat cerebral ischemia/reperfusion injury owing to its suppression of NALP3 inflammasome activation, inhibition of neuronal apoptosis, and attenuation of oxidative stress [30, 31]. In addition, it was found out in some studies that CHR can suppress NF-κB/caspase-1 activation during lipopolysaccharide-induced inflammatory responses in mouse peritoneal macrophages [32, 33]. These findings suggest the possible application of CHR in the treatment of retinal diseases. However, the application of BBH and CHR is limited because of their oxidizability and thermal instability, resulting in low bioavailability.

**Fig. 1:**
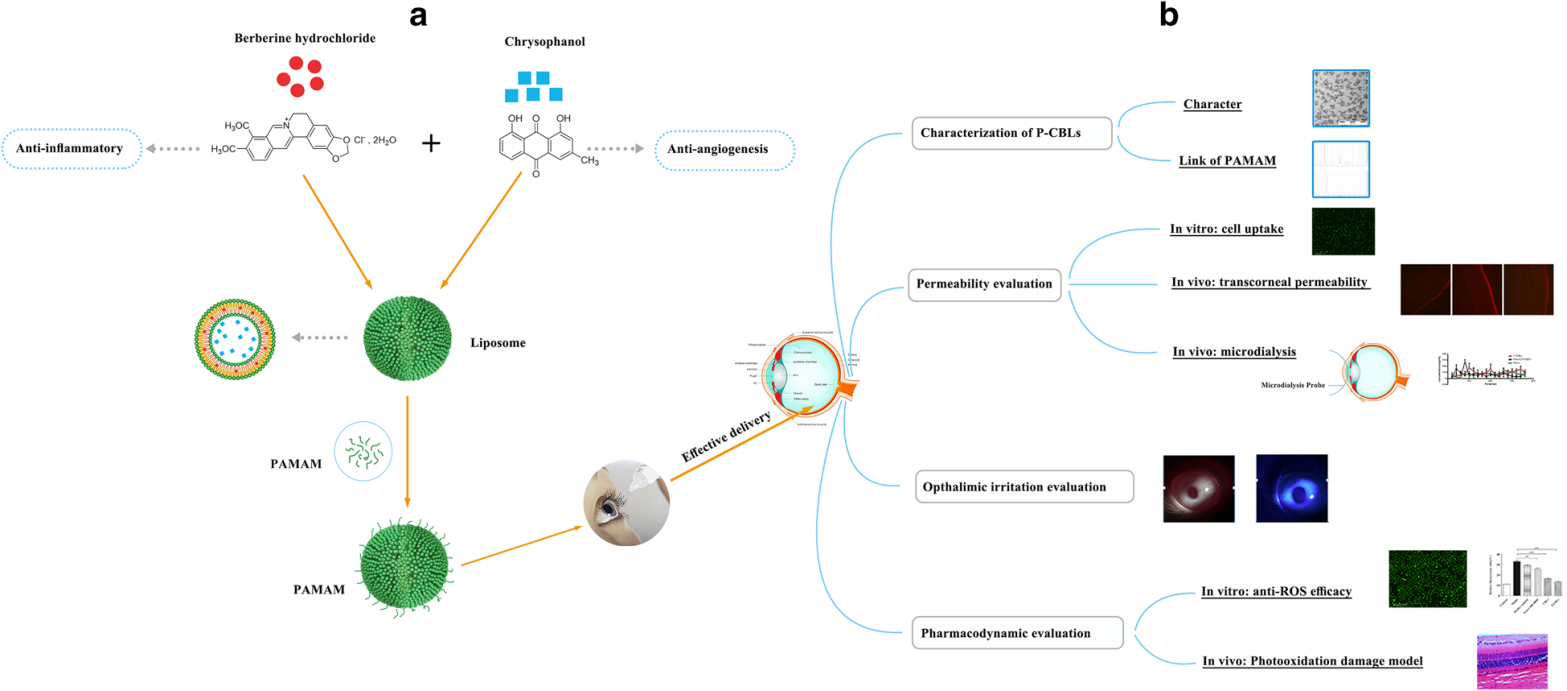
Schematic illustration of the design and evaluation of PAMAM G3.0-coated compound liposomes. **a** Synthesis process of PAMAM coated compound liposomes. Loading BBH and CHR into the different chamber of liposomes by thin film and active load, respectively, and PAMAM G3.0 was loaded into the surface of compound liposomes via electrostatic interaction. **b** Comprehensive evaluation of PAMAM coated compound liposomes including characterization, in vitro, in vivo transport efficiency, preliminary pharmacodynamics studies and opthalmic irritation studies

In this study, we used CHR and BBH as the model drugs for a novel ocular drug-delivery system consisting of PAMAM and liposomes. Cellular uptake, in vivo transcorneal permeability, ocular irritation, and drug absorption after administration were studied in order to clarify whether the PAMAM G3.0-coated compound liposomes were conducive to drug delivery to posterior chamber of eyes. Finally, the therapeutic efficacy was examined via preliminary pharmacodynamics studies including in vitro assessments of anti-reactive oxygen species (ROS) efficacy and protection against photooxidative retinal damage in a light-damaged animal model in comparison with chrysophanol–berberine hydrochloride suspension (CBs), uncoated liposomes, and PAMAM G3.0 liposomes (Fig. 1).

## Results

### Characterization of P-CBLs and CBLs

**Fig. 2:**
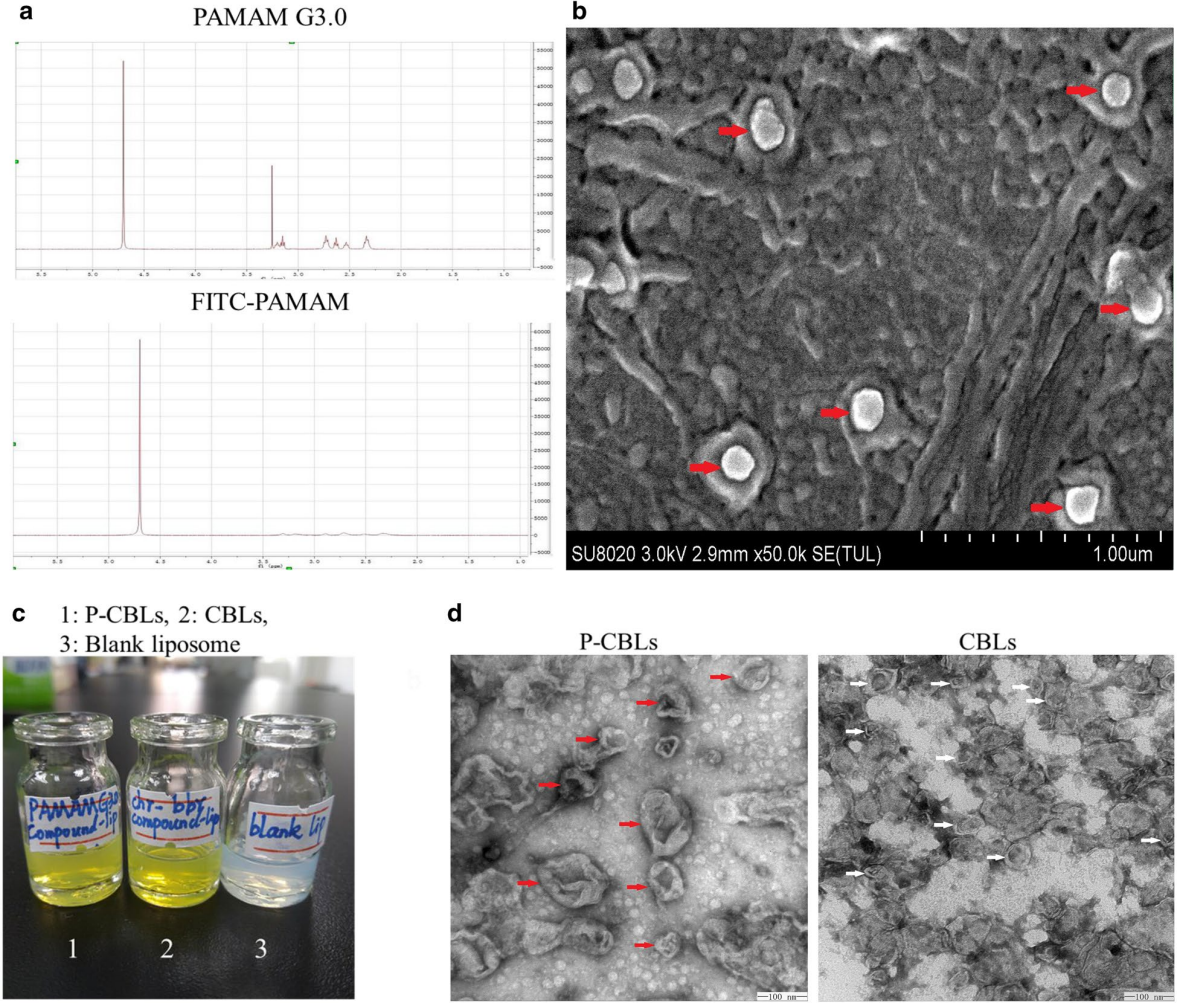
Characterization of FITC-PAMAM coated  liposomes. **a** Verification of FITC onto PAMAM G3.0 via ^1^H-NMR. **b** SEM image of FITC-PAMAM coated liposomes (scale bar = 1 μm). **c** The appearance of CBLs and P-CBLs taken with camera. **d** TEM images of CBLs and P-CBLs, the red arrow indicates the P-CBLs and white indicates CBLs (scale bar = 100 nm)

Fluorescein isothiocyanate (FITC) was grafted onto PAMAM G3.0 via a reaction between the isothiocyanic group of FITC and the NH_2_ termini group of PAMAM G3.0. The comparative proton nuclear magnetic resonance (^1^H-NMR) results before and after the reaction illustrated that the H-signal for the chemical displacement of 2.3–3.3 disappeared (Fig. 2a), indicating that FITC had occupied a C-atom of PAMAM G3.0 successfully. Moreover, as shown in the result, shell with a fine dendritic structure was obviously observed on the surface of CBLs coated with FITC-PAMAM, indirectly proving that PMAMA could coat the CBLs successfully by this method (Fig. 2b). And the fluorescence intensity of FITC-PCBLs was 5.56 × 10^2^.

Corneal permeability is influenced by various factors, including particle size and surface charge [34]. As shown in Table 1, the average sizes of CBLs and P-CBLs were 103.0 ± 18.02 and 148.0 ± 9.98 nm, respectively, indicating that all liposomes were suitable for ocular application and that the average size of P-CBLs was increased because of the coating of the surface of CBLs with PAMAM G3.0. It made no difference of appearance between CBLs and P-CBLs observed with camera (Fig. 2c). The comparative analysis of zeta-potential between CBLs and P-CBLs revealed that the positive potential of CBLs became negative following coating with PAMAM G3.0. CBLs and P-CBLs were uniform and spheroid in shape, without fusion and aggregation (Fig. 2d). Furthermore, the liposomes were coated with a shell (PAMAM), and the particle size increased after being coated. All liposomes were well distributed in the system. The EE% results for CHR and BBH demonstrated that P-CBLs promoted a significant increase of EE%, indicating that PAMAM improved drug loading.

**Table 1:** Characteristic of liposomes formulations (n = 3)

Formulations	Zeta potential (mV)	EE%		PDI
		CHR	BBH	
CBL_S_	6.15 ± 0.78	51.59 ± 3.82	89.6 ± 3.63	0.186 ± 0.027
P-CBLs	− 9.45 ± 1.09	80.69 ± 0.96	93.99 ± 0.05	0.132 ± 0.049

### In vitro cellular internalization

**Fig. 3:**
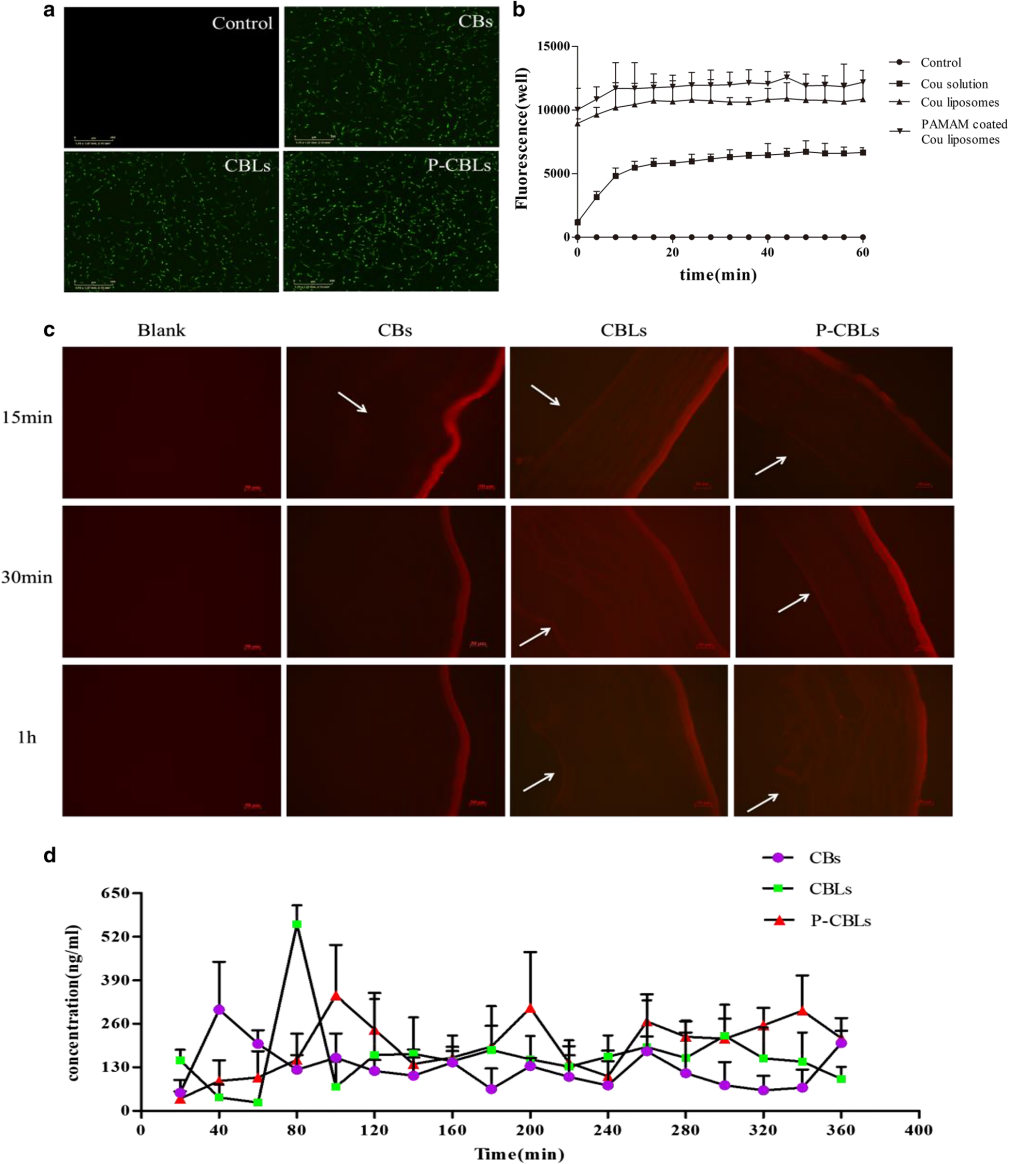
Evaluation of the effectiveness of liposomes delivery. **a** Representative fluorescence images of various formulations containing Cou taken with long-term real-time dynamic live cell imaging analyzer. Following incubated for 24 h and then the cellular uptake of HCECs was captured and analyzed by real-time dynamic monitor (scale bar = 300 μm). **b** Intake count of various formulations. **c** Representative images of different formulation distribution in cornea after administration of respective formulation stained with Nile Red; the arrow indicates the corneal endothelium (scale bar = 50 μm). **d** In vivo pharmacokinetics evaluation following topical administration of various formulations

As shown in Fig. 3a, b, the fluorescence intensity of PAMAM coated coumarin (Cou) liposomes was stronger than that of the other formulations within 1 h after administration, indicating that PAMAM coated Cou liposomes could significantly increase the cellular uptake of therapeutic drug compare with others. The whole process of in vitro cellular internalization for various formulations were showed in Additional files 1, 2, 3, respectively.

### In vivo transcorneal permeability

Results demonstrated that each formulation could permeate the corneal endothelium, and the intensity of fluorescence was similar 15 min after administration. With increasing time after instillation, more CBLs and P-CBLs moved into the corneal epithelium layer, as indicated by the increased fluorescence intensity, and the fluorescence intensity indicated that the formulations were not eliminated by tears. Nevertheless, the fluorescence intensity of CBs in the corneal endothelium was attenuated, suggesting that they could not permeate the corneal epithelium (Fig. 3c).

### In vivo pharmacokinetics

Results revealed that the recovery rates (RR %) were 29.7 ± 6.352% for BBH and 51.42 ± 4.256% for CHR, in line with the quantitative assessment performed via microdialysis. Further, no significant difference of RR % was noted among the different concentrations of CHR and BBH (Additional file 4: Fig. S1A). In addition, the two drugs exhibited good stability for 7 h in in vivo analysis (Additional file 4: Fig. S1B). The BBH concentration in the rabbit aqueous humour of the anterior chamber and time profiles for P-CBLs, CBLs, and CBs after tropical administration were shown in Fig. 3d. P-CBLs and CBLs led to significantly increased aqueous BBH concentrations with peak levels at 100 and 800 min, respectively, whereas the peak of CBs was observed after 40 min. The parameters of the aqueous humour of P-CBLs, CBLs, and CBs were presented in Table 2. The maximum concentrations (*C*_max_) of BBH in the aqueous humour after the administration of P-CBLs and CBLs were 1.719- and 1.23-fold higher, respectively, than those after CBs administration. Meanwhile, the bioactivity of BBH entrapped by liposomes was 1.33-fold higher than that of BBH entrapped by CBs, and P-CBLs increased the bioactivity versus CBs 1.6343 times.

**Table 2:** Main parameters of various formulations after administration in rabbit’s eye (n = 4)

Parameter	CBs	CBLs	P-CBLs
AUC (0–t) (ng/mL)	40,780.94 ± 9715.737	54,294.446 ± 13,066.52	67,033.93 ± 9105.516**
AUC (0–∞) (ng/mL)	43,825.184 ± 10,967.05	61,824 ± 45,069.22	12,314.38 ± 69,431.46*
Tmax (min)	45 ± 10	80	130 ± 47.61
Cmax (ng/mL)	323.9 ± 121.4	556.895 ± 56.859*	400.023 ± 108.757*^#^
t1/2z	131.442 ± 162.889	2326.416 ± 4563.18	241.006 ± 168.415

* *P* < 0.05, ** *P* < 0.01, compared with CBs; # *P* < 0.05, compared with CBLs

### Preliminary pharmacodynamics studies

#### Protection against photooxidative retinal damage

**Fig. 4:**
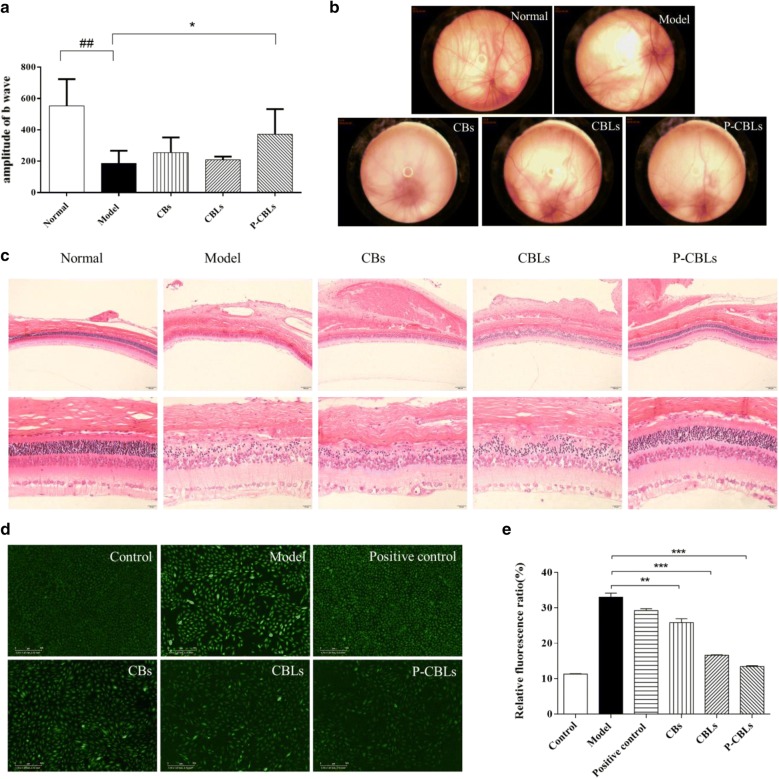
Protection against photooxidative retinal damage. **a** Change of b-wave amplitude following topical administration of respective formulations for consecutive 14 days. **b** Fundus retinography of various formulations. **c** The protective efficacy evaluation of various formulations in the retina by staining hematoxylin and eosin (H&E) (scale bar = 20 μm). **d** Photographs of in vitro anti-ROS efficacy taken with a long-term real-time dynamic live cell imaging analyzer. **e** ROS level of various formulations. Data were presented as the mean ± SD (n = 3). **P < 0.01, ***P < 0.001

P-CBLs induced the greatest protection of retinal function in light-exposed rats. Flash electroretinograms (FERGs) were recorded on day 14 after light damage, demonstrating significantly increased b-wave responses in P-CBLs-treated rats compared with the findings in the other groups (Fig. 4a).

The retinal vessels of rats in the normal saline group were intact, and the background of the fundus was clearly displayed. There were no abnormal changes in the vessels of rats in the blank liposomes group, but the reflection of the fundus was particularly severe. P-CBLs had no effect on the blood vessels, but they improved the large area of reflection under the eyes (Fig. 4b).

Compared with the findings in the normal saline group, the outer nuclear layer in the blank liposomes group became thinner, and the number of outer nuclear layer cells decreased significantly. The histopathological findings of sections through the eye revealed obvious protective effects in the retina in P-CBLs-treated rats after photooxidative retinal injury, in contrast with the findings in blank-liposome-exposed rats. Morphometric analysis indicated that P-CBLs-treated rats had clear layers of retinal structures, including neatly arrayed and clearly stained outer nuclear layers (Fig. 4c).

#### In vitro antioxidant efficacy

Figure 4d, e revealed that CHR and BBH could reduce intracellular ROS levels effectively compared with the findings in the model group, and P-CBLs was the most potent according to their relative fluorescence ratio (fluorescence confluence/cell confluence). The process of in vitro anti-ROS for various formulations were shown in Additional files 5, 6, 7, 8, respectively.

### Ophthalmic irritation studies

**Fig. 5:**
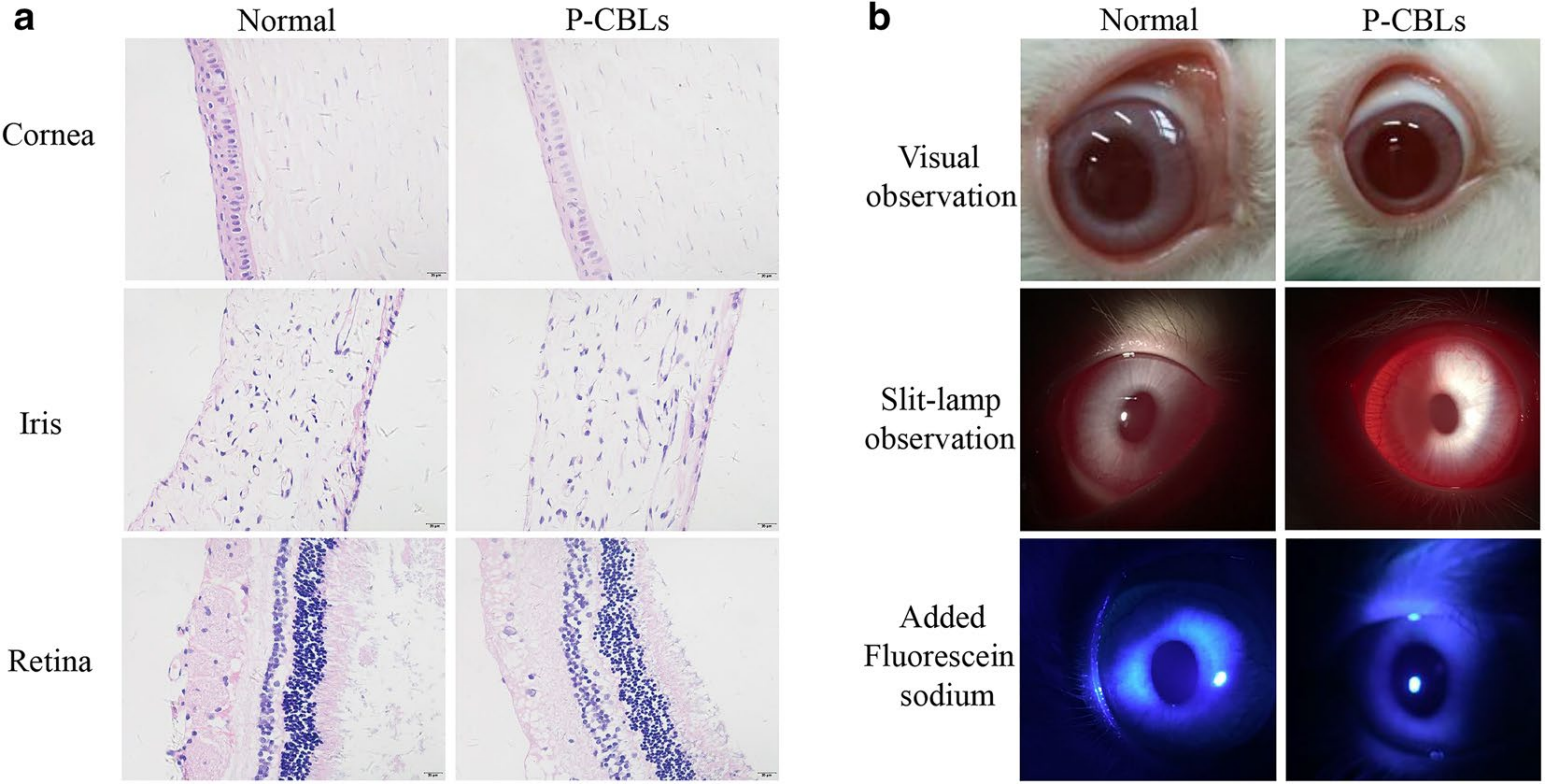
Ophthalmic irritation studies. **a** Representative images of the tissue histology after P-CBLs instillation for 14 consecutive days (scale bar = 20 μm). **b** Observation of ocular surface using a silt lamp and camera, stained with 0.5% sodium fluorescein

The representative images of the tissue histology after P-CBLs instillation for 14 consecutive days were shown in Fig. 5a, and no obvious injury or abnormality was observed in the cornea, iris, or conjunctiva. Further, Fig. 5b showed that no injury and edema occurred in the ocular surface, as observed using a silt lamp and camera, and staining with 0.5% sodium fluorescein further confirmed the safety of P-CBLs.

## Discussion

In our study, BBH and CHR were chosen as the therapeutic agents for AMD because of their diverse pharmacological activities. Nevertheless, it is difficult to encapsulate two drugs with different polarities. In previous studies, it was observed that precipitation occurred when CHR and BBH were encapsulated at a mass ratio of 1:1 (Lec/CHR = 25:1, mass ratio), affecting the drug loading capacity. In order to address these problems, we prepared CHR liposomes (Lec/CHR = 60:1, mass ratio) via thin-film dispersion and the entry of BBH (Lec/BBH = 25:1, mass ratio) by adjusting the pH for active loading, leading to an acceptable encapsulated concentration with an EE% of 51.59 for CHR and 89.6 for BBH. Further, in this study, the results also indicated that PAMAM G3.0 greatly contributed to improved encapsulation efficiency. The possible interaction occurred between the drug molecule and abundant groups formed via electrostatic interactions in accordance with Devarakonda et al. [35].

In vitro and in vivo transport efficiency studies represented a pivotal strategy for evaluating ocular drug delivery. In our study, transport efficiency was evaluated from three aspects, namely, cellular internalization, transcorneal permeability, and pharmacokinetics. First, cellular internalization for a preliminary targeting study was evaluated in HCECs, and Cou [36] was chosen because neither CHR nor BBH exhibited fluorescence, indirectly evaluating the cellular internalization ability for various formulations. Previously, laser-scanning confocal microscopy has been the most commonly used strategy for assessing the extent of cellular uptake. Nevertheless, the clear dynamic process of cellular uptake could not be observed in real time by this approach. In this study, we adopted a long-term real-time dynamic live cell imaging analyzer in order to record the status continuously at each time point. The results indicated that the coated liposomes delivered drugs quickly with a high efficiency.

Secondly, transcorneal permeability was adopted in order to evaluate the bioadhesion of various formulations, providing a preliminary evaluation of drug bioavailability. The result demonstrated that P-CBLs was more efficient in transporting Cou than CBLs. Conversely, CBs provided minimal uptake into cells, and a higher degree of fluorescence in deep layers of the cornea occurred using liposome formulations and the most of P-CBLs. Although numerous studies illustrated that small, positively charged nanoparticles induced better accumulation in the cornea than negatively charged nanoparticles on the basis of electrostatic interactions with negatively charged cellular components, an amino group in PAMAM G3.0 possessed mucoadhesive properties with the negatively charged cellular membrane in accordance with Souza et al. [37], and the diffusion of PAMAM G3.0 occurred through the sclera, allowing greater permeability of negatively charged molecules than of positively charged molecules into corneal layers [38].

Meanwhile, pharmacokinetic studies were further applied to examine the transport efficiency of P-CBLs. Challenges remained in evaluating drug deposition because of the anatomical structure and size of the eye. Recently, ocular microdialysis has been gradually employed owing to its ability to continuously monitor drug disposition and overcome the problem of high variability by reducing the need for animal models [39]. Our findings demonstrated that CBLs and P-CBLs significantly enhanced the solubility and ocular absorption of BBH. Furthermore, in the present study, the half-life of BBH inside the anterior chamber could be prolonged by liposome-entrapped PAMAM G3.0. However, we could not detect CHR using HPLC–MS–MS at concentrations below 100 ng/mL primarily owing to its phenolic structure. Further, no quantitative assessment of CHR could be performed even though the drug and its metabolites were detected via HPLC and HPLC–MS–MS. There are few literature reports of the quantitative assessment of CHR. Therefore, the LC–MS–MS methodology was only established for BBH for use in in vivo pharmacokinetics studies.

Therapeutic efficacy and safety were further examined to validate our hypothesis. We investigated the therapeutic effects of the drugs against AMD in vitro and in vivo. First, in vitro antioxidant efficacy was examined in RPE-19 cells exposed to H_2_O_2_, and then we evaluated the efficacy of the drugs according to the fluorescence intensity to calculate ROS levels. Second, the in vivo therapeutic efficacy was analyzed in Sprague–Dawley rats in order to further confirm their potential for treating AMD. Different animal models, including laser-, light-, and sodium-iodide-induced retinal degeneration models, are currently available for pharmacodynamic analysis [40]. Among these different models, the laser-induced model has been commonly employed in clinical research because it can induce CNV successfully. However, some classical features of AMD (drusen, retinal inflammation) are not established in this model. Although some significant hallmark features such as drusen and CNV were not induced by light damage, macrophage accumulation was induced effectively in addition to outer retinal atrophy [41, 42], and retinal degeneration associated with light damage was similar to the natural findings [43]. Thus, the therapeutic efficacy in our experiment was investigated using a light-induced model. The results illustrated the therapeutic efficacy of CHR and BBH in reducing ROS levels and protecting against light-induced retinal damage. Furthermore, we also observed that P-CBLs effectively promoted CHR and BBH accumulation, achieving a better therapeutic effect.

The data presented in our study has provided preliminary evidence supporting our hypothesis, but exploration of the pharmacokinetics of CHR using advanced techniques and molecular mechanisms for protecting against photooxidation should be conducted in future work. Meanwhile, another limitation of our study was that the preliminary pharmacodynamics studies were only performed in a photooxidative model, which is not representative of the multiple mechanisms involved in AMD. Thus, different models associated with AMD should be assessed in order to evaluate the therapeutic efficacy of CHR and BBH more comprehensively.

In conclusion, our findings suggested that P-CBLs could reach the target tissues and achieve appreciable therapeutic efficacy against photooxidative stress based on the various pharmacologic activities of CHR and BBH, providing a new strategy for achieving novel ocular drug delivery with compound agents for AMD therapy.

## Materials and methods

### Materials

Lecithin (Lec, soybean, > 98%), cholesterol (Chol, AR > 95%), dl-α-tocopherol, and octadecylamine were supplied by Aladdin Industrial Co., Ltd. (Shanghai, China). PAMAM G3.0 dendrimer (ethylenediamine core, generation 3.0 solution) was obtained from Sigma-Aldrich (St. Louis, MO, USA). CHR (> 98% purity) and BBH (> 98% purity) were purchased from Sichuan Weikeqi Biological Technology Co., Ltd. (Chengdu, China). FITC was purchased from Meilunbio Co., Ltd. (Dalian, Liaoning, China). All other chemicals and reagents were of analytical grade and were used as received.

### Animals and cells

New Zealand white rabbits (weighing 2.0–2.5 kg) and male Sprague–Dawley rats (weighing 210–250 g) were purchased from the Laboratory Animal Center of Guangzhou University of Chinese Medicine.

HCECs (human corneal epithelial cells) were kindly donated by the Sun Yat-sen University Ophthalmology Center Hospital. Human retinal pigment epithelial cells (ARPE-19) were obtained from the Cell Bank of the Chinese Academy of Sciences (Wuhan, China).

### Preparation of compound liposomes (CBLs)

Briefly, CBLs were prepared via two steps. First, Lec/Chol/CHR, dl-α-tocopherol, and octadecyl amine (80:20:1, mass ratio) were dissolved in anhydrous ethanol and then evaporated using a rotary evaporator (Yamato, Japan) at 60 °C under reduced pressure to produce the thin film. Then, the sample was dried at room temperature for 2 h and then hydrated with 10 mL of sodium citrate buffer (pH = 3.47) at 40 °C to induce phase transition. The CHR liposome suspensions were sonicated at 200 W for 3.3 min and filtered using a 0.22 μm microporous membrane filter to achieve the desired size. Then, the sorted CHR liposomes were added to a 0.4 mg/mL BBH solution at a volume mass ratio of 1:1 (Lec/BBH = 25:1, mass ratio), and then the pH of the mixture was adjusted to 7.0, followed by incubation in a water bath at 60 °C for 20 min to produce the CHR-BBH compound liposomes. The drug concentration of BBH was 0.1600 mg/mL and 0.0500 mg/mL of CHR in CBLs.

### Labeling of FITC onto PAMAM G3.0

Because the structure of PAMAM G3.0 has no fluorescence or ultraviolet absorption, it is difficult to detect it qualitatively or quantitatively by conventional methods. However, FITC-PAMAM has good fluorescence characteristics and can be generated by the reaction of the amino group in PAMAM G3.0 with FITC’s sulfur cyanide as a qualitative or quantitative method for PAMAM G3.0.

According to the pilot studies [44], PAMAM G3.0 (139.9 mg) was dissolved in 2 mL of methanol and then slowly added to 5 mL of FITC (9.46 mg, PAMAM/FITC = 1:1.2, molar ratio) methanol solution. The mixture was stirred overnight at room temperature until the methanol volatized. The residual solid was dissolved in distilled water and then dialyzed for 4 days. Then, FITC-PAMAM was obtained via lyophilization, and verification of successful synthesis was performed via ^1^H-NMR (Bruker, USA).

### Preparation of FITC-PAMAM coated compound liposomes or PAMAM coated compound liposomes (P-CBLs) and characterization of P-CBLs

Freeze-dried FITC was redissolved in distilled water (0.5 mg/mL) and added slowly to CBLs, followed by stirring at room temperature for 5 h (at a molar ratio of 0.2%). Then, the sample was dialyzed with distilled water for 3 days to remove free FITC-PAMAM. A transmission electron microscope (TEM, HITACHI SU8020, Japan) was used to verify whether FITC-PAMAM entrapped the liposomes successfully. Then, P-CBLs were produced according to a previously described method, and the size distribution and zeta-potential before and after coating were compared using Malvern Instruments Zetasizer HS III (Malvern, UK). The morphology of CBLs and P-CBLs was observed using a scanning electron microscope (SEM, JEOL Jem-1010, Japan). The encapsulation efficiency (EE%) of CBLs and P-CBLs were analyzed by HPLC (Additional file 9, Shimadzu, Japan). The drug concentration of BBH was 0.1488 mg/mL and 0.0465 mg/mL of CHR in P-CBLs, respectively.

### In vitro cellular internalization

HCECs was cultured in accordance with a previous study by Liu et al. [45]. The cellular internalization of various formulations on the viability of HCECs were studied by long-term real-time dynamic live cell imaging analyzer.(Essen BioScience, Inc.) The dynamic process of cellular internalization was photographed within 1 h to observe cellular uptake after incubated with various formulations containing Cou for 24 h.

Cou liposomes were prepared by thin-film dispersion method that as the same as the CHR liposome (mentioned in “Preparation of compound liposomes (CBLs)” section). Then, the PAMAM coated Cou liposomes were prepared by the same method as P-CBLs. The final concentration of Cou was 0.2737 μM for Cou liposomes and 0.2390 μM for PAMAM coated cou liposomes, respectively.

### In vivo transcorneal permeability

CBs, CBLs, and P-CBLs were used to investigate in vitro transcorneal permeability, and all formulations were stained with Nile Red in order to evaluate corneal permeability due to CHR and BBH did not exhibit fluorescence. The drug concentrations of CBLs and P-CBLs were the same as mention in “Preparation of compound liposomes (CBLs)” and “Preparation of FITC-PAMAM compound liposomes or PAMAM compound liposomes (P-CBLs) and characterization of P-CBLs”. The drug concentration of CBs was the same as CBLs group. All rabbits were given 50 μL formulations into the lower conjunctival sac after anesthesia with 3% pentobarbital sodium solution, followed by sacrificed to excise the corneal tissue after 15, 30, and 60 min. The tissue was stained with hematoxylin and eosin (H&E) to observe permeability in the deeper corneal endothelial layer using an inverted fluorescence microscope (Phenix, China).

### In vivo pharmacokinetics

Normal saline containing 30% ethanol was chosen as the perfusate for two drugs with opposing polarity, and the flow rate was 1.5 μL/min according to our previous study. Then, an in vitro RR% evaluation was performed via dialysis and retrodialysis. The in vivo stability study was performed using a retrodialysis method, which provided the evidence for the microdialysis method that was used for the in vivo pharmacokinetics study.

New Zealand rabbits were used to investigate the in vivo pharmacokinetics regarding transport efficiency using a microdialysis technique. Normal saline containing 30% ethanol was chosen as the perfusate for two drugs with opposing polarity, and the flow rate was 1.5 μL/min according to our previous study. A liner probe (CMA 30, sweden) was inserted into the ocular anterior chamber of each animal to collect samples after anesthesia with a 3% pentobarbital sodium solution and continuous anesthesia throughout the experimental period. The drug concentrations of CBs, CBLs, and P-CBLs were the same as before. Then, 50 μL of the formulations was administered into the lower conjunctival sac of the right eye of each rabbit using a micropipette after recovery of the anterior aqueous humour, and samples (30 μL) were collected over a 20 min period starting 6 h after instillation and stored at − 20 °C. Samples were analyzed by HPLC–MS–MS (Agilent, USA), and data were obtained to fit the concentration–time curve and then analyzed using Drug and Statistics Software (DAS 3.0) to calculate the pharmacokinetic parameters of each formulation.

### Preliminary pharmacodynamics studies

#### Protection against photooxidative retinal damage

The therapeutic efficacy and ocular transport efficiency of liposome formulations were investigated using a light-induced damaged model of retinal inflammation. Male Sprague–Dawley rats underwent visual electrophysiology after the intraperitoneal injection of 3% pentobarbital sodium and assigned to five groups randomly according to the value of b-wave amplitude as follows: normal saline, model (blank liposomes), CBs, CBLs, and P-CBLs groups.

All animals were exposed to 16,000 ± 1500 lux light for 8 h, excluding animals in the normal saline group. During light damage, animals were placed in a special illumination cage held at a constant temperature, with free access to food and water. After light damage, animals in the five groups were given treatments into both eyes three times a day for 14 consecutive days. The drug concentrations of CBs, CBLs, and P-CBLs were the same as before. And then animals were given 10 μL formulations for each eye.

All animals were monitored via flash electroretinography using a fundus camera, and the retinas were excised following euthanasia via an overdose of pentobarbital. H&E staining was used to evaluate the protective efficacy of P-CBLs in the retina.

#### In vitro anti-ROS efficacy

Human retinal pigment epithelial cells (ARPE-19) were maintained in Dulbecco’s modified Eagle’s medium/Ham’s F12 (Gibco) supplemented with 10% fetal bovine serum (Gibco), 100 IU/mL penicillin (Gibco), and 100 μg/mL streptomycin (Gibco) at 37 °C in an atmosphere of 5% CO_2_. After incubation, cells were exposed to 200 μM H_2_O_2_ for 24 h, and fluorescent probes (Beyotime Biotechnology, China) were used to measure intracellular ROS levels (DCFH-DA, 10 mM) according to the manufacturer’s instructions. Before application, CBLs and P-CBLs of a certain volume were diluted with complete culture medium. The final concentration of BBH was 0.0040 μM and 0.0020 μM for CHR in CBLs groups, while 0.0044 μM of BBH, 0.0018 μM for CHR for P-CBLs, respectively. The final drug concentration of CBs was the same as CBLs group. Treated cells were loaded with fluorescent probes in a serum-free medium for 20 min at 37 °C. Then, photographs were taken using a long-term real-time dynamic live cell imaging analyzer.

### Ophthalmic irritation studies

#### Single-dose irritation test

New Zealand rabbits that were free of inflammation and ocular injury were divided into groups and given 50 μL of various formulations of the treatments to the right side of the eyes, whereas the left side was treated with normal saline as the control. The drug concentrations of CBs, CBLs, and P-CBLs were the same as before. The ocular surface structure was evaluated at 1, 4, 8, and 24 h using the Draize eye test.

#### Long-term irritation test

Rabbits were given P-CBLs three times once daily for 14 consecutive days in accordance with the single-dose irritation test, and all animals were monitored closely using a slit lamp and a camera. The eyes of the animals were also exposed to 0.5% sodium fluorescein, and the staining was scored using the Draize eye test throughout the experiment. Further, histological analysis was performed in order to further examine the safety of P-CBLs in different tissues around the anterior chamber after 14 days of treatment. Tissues were stained with H&E and then observed using a microscope (Olympus, Japan).

### Statistical analysis

Data were presented as the mean ± standard deviation (SD) from at least three independent experiments. Statistical analyses were performed using SPSS 20.0 software via one-way analysis of variance (ANOVA) (*n* ≥ 3, *α* = 0.05), and the obtained pharmacokinetic data were analyzed using DAS 3.0 software. A value of *P *< 0.05 indicated statistical significance.

## Additional files

**Additional file 1.** Video of CBs for in vitro cellular internalization.

**Additional file 2.** Video of CBLs for in vitro cellular internalization.

**Additional file 3.** Video of P-CBLs in vitro cellular internalization.

**Additional file 4: Figure S1.** Methodology examination of ocular microdialysis. **A** Recovery rates of microdialysis probes at different concentrations. **B** Recovery rates and recovery loss of microdialysis probes in 7 h. RR%: Recovery rates, RL%: Recovery loss.

**Additional file 5.** Video of model group for in vitro anti-ROS efficacy.

**Additional file 6.** Video of CBs for in vitro anti-ROS efficacy.

**Additional file 7.** Video of CBLs for in vitro anti-ROS efficacy.

**Additional file 8.** Video of P-CBLs for in vitro anti-ROS efficacy.

**Additional file 9.** HPLC analysis of the P-CBLs encapsulation efficiency (EE%).

## Data Availability

All data and material are included in the article and its additional files.

## Abbreviations

AMD: age-related degeneration
BBH: berberine hydrochloride
CHR: chrysophanol
PAMAM G3.0: the third-generation polyamidoamine dendrimer
CBLs: compound liposomes
P-CBLs: PAMAM coated compound liposomes
CBs: chrysophanol–berberine hydrochloride suspension
RR%: recovery rates
Cou: coumarin
Lec: Lecithin
Chol: cholesterol
HCECs: human corneal epithelial cells
ARPE-19: human retinal pigment epithelial cells
TEM: transmission electron microscope
SEM: scanning electron microscope
EE%: encapsulation efficiency
H&E: hematoxylin and eosin
ROS: reactive oxygen species

## References

[1] Gordois A, Cutler H, Pezzullo L, Gordon K, Cruess A, Winyard S (2012). An estimation of the worldwide economic and health burden of visual impairment. Glob Public Health..

[2] Wong WL, Su X, Li X, Cheung CM, Klein R, Cheng CY (2014). Global prevalence of age-related macular degeneration and disease burden projection for 2020 and 2040: a systematic review and meta-analysis. Lancet Glob Health..

[3] Group TC (2011). Ranibizumab and bevacizumab for neovascular age-related macular degeneration. N Engl J Med.

[4] Ip MS (2004). Intravitreal injection of triamcinolone: an emerging treatment for diabetic macular edema. Diabetes Care.

[5] Jonas JB, Spandau UH, Schlichtenbrede F (2008). Short-term complications of intravitreal injections of triamcinolone and bevacizumab. Eye.

[6] Cunningham M, Edelman JS (2008). Intravitreal steroids for macular edema: the past, the present, and the future. Surv Ophthalmol..

[7] Falavarjani KG, Nguyen QD (2013). Adverse events and complications associated with intravitreal injection of anti-VEGF agents: a review of literature. Eye.

[8] Wang X, Sawada T, Sawada O, Saishin Y, Liu P, Ohji M (2014). Serum and plasma vascular endothelial growth factor concentrations before and after intravitreal injection of aflibercept or ranibizumab for age-related macular degeneration. Am J Ophthalmol.

[9] Ragelle H, Riva R, Vandermeulen G, Naeye B, Pourcelle V, Le DC (2014). Chitosan nanoparticles for siRNA delivery: optimizing formulation to increase stability and efficiency. J Control Release.

[10] Guo C, Zhang Y, Yang Z, Li M, Li F, Cui F (2015). Nanomicelle formulation for topical delivery of cyclosporine A into the cornea: in vitro mechanism and in vivo permeation evaluation. Sci Rep.

[11] Mastorakos P, Kambhampati SP, Mishra MK, Wu T, Song E, Hanes J (2015). Hydroxyl PAMAM dendrimer-based gene vectors for transgene delivery to human retinal pigment epithelial cells. Nanoscale..

[12] Diebold Y, Jarrín M, Sáez V, Carvalho EL, Orea M, Calonge M (2007). Ocular drug delivery by liposome-chitosan nanoparticle complexes (LCS-NP). Biomaterials.

[13] Bochot A, Fattal E, Boutet V, Deverre JR, Jeanny JC, Chacun H (2002). Intravitreal delivery of oligonucleotides by sterically stabilized liposomes. Investig Ophthalmol Vis Sci..

[14] Ravar F, Saadat E, Gholami M, Dehghankelishadi P, Mahdavi M, Azami S (2016). Hyaluronic acid-coated liposomes for targeted delivery of paclitaxel, in vitro characterization and in vivo evaluation. J Control Release.

[15] Wang JL, Liu YL, Li Y, Dai WB, Guo ZM, Wang ZH (2012). EphA2 targeted doxorubicin stealth liposomes as a therapy system for choroidal neovascularization in rats. Investig Ophthalmol Vis Sci..

[16] Wang WY, Yao C, Shao YF, Mu HJ, Sun KX (2011). Determination of puerarin in rabbit aqueous humor by liquid chromatography tandem mass spectrometry using microdialysis sampling after topical administration of puerarin PAMAM dendrimer complex. J Pharm Biomed Anal.

[17] Choi SK, Thomas T, Li MH, Kotlyar A, Desai A, Baker JR (2010). Light-controlled release of caged doxorubicin from folate receptor-targeting PAMAM dendrimer nanoconjugate. Chem Commun.

[18] Biswas S, Deshpande PP, Navarro G, Dodwadkar NS, Torchilin VP (2013). Lipid modified triblock PAMAM-based nanocarriers for siRNA drug co-delivery. Biomaterials.

[19] Morenobondi MC, Orellana G, Turro NJ, Tomalia DA (1990). Photoinduced electron-transfer reactions to probe the structure of starburst dendrimers. Macromolecules.

[20] Gopidas KR, Leheny AR, Caminati G, Turro NJ, Tomalia DA (1991). Photophysical investigation of similarities between starburst dendrimers and anionic micelles. J Am Chem Soc.

[21] Jansen JFGA, Meijer EW (1994). Encapsulation of guest molecules into a dendritic box. Science.

[22] Jansen JFGA, Meijer EW (1995). The dendritic box: shape-selective liberation of encapsulated guests. J Am Chem Soc.

[23] Ribeiro RA, Limajunior RCP, Leite CAVG, Mota JMSC (2012). Chemotherapy-induced hemorrhagic cystitis: pathogenesis, pharmacological approaches and new insights. J Exp Integr Med.

[24] Choi YH (2016). Berberine hydrochloride protects C2C12 myoblast cells against oxidative stress-induced damage via induction of Nrf-2-mediated HO-1 expression. Drug Dev Res.

[25] Zhang Y, Li X, Zhang Q, Li J, Ju J, Du N (2014). Berberine hydrochloride prevents postsurgery intestinal adhesion and inflammation in rats. J Pharmacol Exp Ther.

[26] Wen SQ, Jeyakkumar P, Avula SR, Zhang L, Zhou CH (2016). Discovery of novel berberine imidazoles as safe antimicrobial agents by down regulating ROS generation. Bioorg Med Chem Lett.

[27] Pang B, Zhao LH, Zhou Q, Zhao TY, Wang H, Gu CJ (2015). Application of berberine on treating type 2 diabetes mellitus. Int J Endocrinol.

[28] Yin J, Ye J, Jia W (2012). Effects and mechanisms of berberine in diabetes treatment. Acta Pharmaceutica Sinica B.

[29] Tanabe H, Suzuki H, Nagatsu A, Mizukami H, Ogihara Y, Inoue M (2006). Selective inhibition of vascular smooth muscle cell proliferation by coptisine isolated from Coptis rhizoma, one of the crude drugs composing Kampo medicines Unsei-in. Phytomedicine.

[30] Zhang N, Zhang X, Liu X, Wang H, Xue J, Yu J (2014). Chrysophanol inhibits NALP3 inflammasome activation and ameliorates cerebral ischemia/reperfusion in mice. Mediat Inflamm.

[31] Yan J, Zheng M, Zhang D (2014). Chrysophanol liposome preconditioning protects against cerebral ischemia–reperfusion injury by inhibiting oxidative stress and apoptosis in mice. Int J Pharmacol.

[32] Kim SJ, Kim MC, Lee BJ, Park DH, Hong SH, Um JY (2010). Anti-inflammatory activity of chrysophanol through the suppression of NF-kappaB/caspase-1 activation in vitro and in vivo. Molecules.

[33] Lin F, Zhang C, Chen X, Song E, Sun S, Chen M (2015). Chrysophanol affords neuroprotection against microglial activation and free radical-mediated oxidative damage in BV2 murine microglia. Int J Clin Exp Med.

[34] Fujisawa T, Miyai H, Hironaka K, Tsukamoto T, Tahara K, Tozuka Y (2012). Liposomal diclofenac eye drop formulations targeting the retina: formulation stability improvement using surface modification of liposomes. Int J Pharm.

[35] Buczkowski A, Waliszewski D, Urbaniak P, Palecz B (2016). Study of the interactions of PAMAM G3-NH 2 and G3-OH dendrimers with 5-fluorouracil in aqueous solutions. Int J Pharm.

[36] Hironaka K, Inokuchi Y, Tozuka Y, Shimazawa M, Hara H, Takeuchi H (2009). Design and evaluation of a liposomal delivery system targeting the posterior segment of the eye. J Control Release.

[37] Souza JG, Dias K, Silva SAM, Rezende LCDD, Rocha EM, Emery FS (2015). Transcorneal iontophoresis of dendrimers: PAMAM corneal penetration and dexamethasone delivery. J Control Release.

[38] Maurice DM, Polgar J (1977). Diffusion across the sclera. Exp Eye Res.

[39] Boddu SH, Gunda S, Earla R, Mitra AK (2010). Ocular microdialysis: a continuous sampling technique to study pharmacokinetics and pharmacodynamics in the eye. Bioanalysis..

[40] Zeiss CJ (2010). Animals as models of age-related macular degeneration: an imperfect measure of the truth. Vet Pathol.

[41] Fernando N, Natoli R, Valter K, Provis J, Rutar M (2016). The broad-spectrum chemokine inhibitor NR58-3.14.3 modulates macrophage-mediated inflammation in the diseased retina. J Neuroinflamm.

[42] Rutar M, Natoli R, Chia RX, Valter K, Provis JM (2015). Chemokine-mediated inflammation in the degenerating retina is coordinated by Müller cells, activated microglia, and retinal pigment epithelium. J Neuroinflamm.

[43] Lin FL, Lin CH, Ho JD, Yen JL, Chang HM, Chiou GCY (2017). The natural retinoprotectant chrysophanol attenuated photoreceptor cell apoptosis in an *N*-methyl-*N*-nitrosourea-induced mouse model of retinal degenaration. Sci Rep.

[44] Shen Y, Zhou Z, Sui M, Tang J, Xu P, Van Kirk EA (2010). Charge-reversal polyamidoamine dendrimer for cascade nuclear drug delivery. Nanomedicine..

[45] Liu J, Song G, Wang Z, Huang B, Gao Q, Liu B (2007). Establishment of a corneal epithelial cell line spontaneously derived from human limbal cells. Exp Eye Res.

