# Supplementary material for: Liposomes for effective drug delivery to the ocular posterior chamber
Source: J Nanobiotechnology. 2019 May 13;17:64. doi: 10.1186/s12951-019-0498-7 (PMC6515668; doi:10.1186/s12951-019-0498-7)
Supplement: Supplementary file 4 — Additional file 4: Figure S1. Methodology examination of ocular microdialysis. A Recovery rates of microdialysis probes at different concentrations. B Recovery rates and recovery loss of microdialysis probes in 7 h. RR%: Recovery rates, RL%: Recovery loss. [file 12951_2019_498_MOESM4_ESM.docx]

**
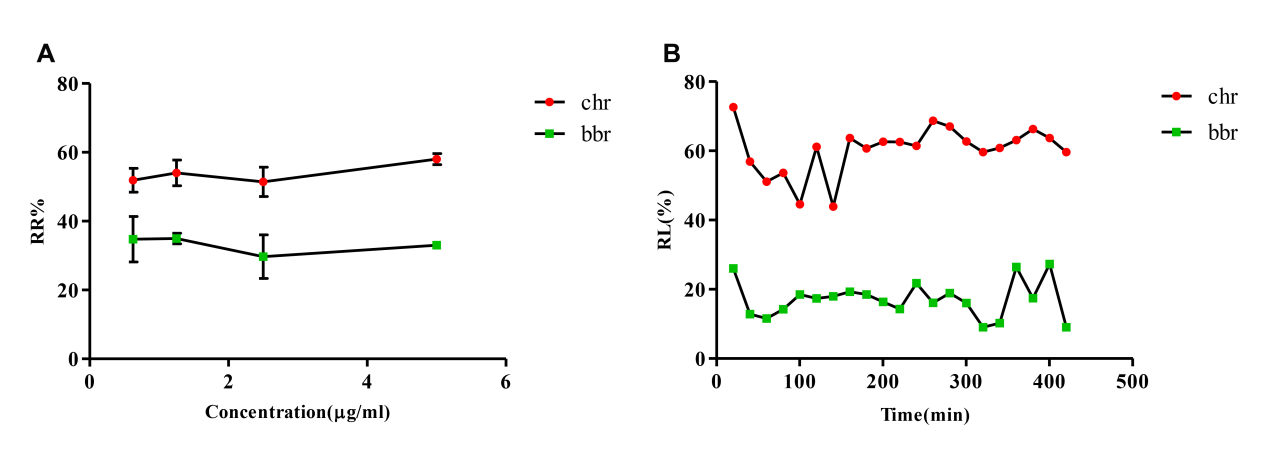
**

**Additional File 4：Figure S1** Methodology examination of ocular microdialysis. **A)** Recovery rates of microdialysis probes at different concentrations. **B)** Recovery rates and recovery loss of microdialysis probes in 7 h. RR%: Recovery rate, RL%: Recovery loss
