# Supplementary material for: Liposomes for effective drug delivery to the ocular posterior chamber
Source: J Nanobiotechnology. 2019 May 13;17:64. doi: 10.1186/s12951-019-0498-7 (PMC6515668; doi:10.1186/s12951-019-0498-7)
Supplement: Supplementary file 9 — Additional file 9. HPLC analysis of the P-CBLs encapsulation efficiency (EE%). [file 12951_2019_498_MOESM9_ESM.pdf]

HPLC Analysis of the P-CBLs Encapsulation Efficiency(EE%)

In order to determine the EE% of two compositions of P-CBLs, CHR was measured using protamine-based chromatography, whereas Sephadex chromatography was used for BBH. Briefly, P-CBLs (0.5 mL) were mixed with 10 mg/mL protamine, reacted for 3 min at room temperature, diluted with normal saline to 5 mL, and centrifuged at 3,000 rpm for 30 min to obtain the supernatant for analysis. For BBH, P-CBLs (0.5 mL) were applied to the column, and the eluent was collected. All samples were dissolved in methanol and injected at a volume of 20 μL into a Kromasil 100-5-C18 column (250 mm × 4.6 mm, 5 μm), and 0.1% phosphoric acid–methanol served as the mobile phase with a gradient elution. The flow rate was 1 mL/min and the detection was performed constantly at 35 ℃.
